# P-1478. Assessment of Beta-lactam Susceptibility Profiles in *Pseudomonas aeruginosa*: A Comprehensive Analysis at an Academic Medical Center

**DOI:** 10.1093/ofid/ofae631.1648

**Published:** 2025-01-29

**Authors:** David S Burgess, Katie B Olney, Donna R Burgess

**Affiliations:** University of Kentucky College of Pharmacy, Lexington, Kentucky; University of Kentucky HealthCare, Lexington, Kentucky; UK HealthCare, Lexington, KY

## Abstract

**Background:**

*Pseudomonas aeruginosa* is one of the most difficult bacterial pathogens to treat. As beta-lactams remain the most prescribed class of antimicrobials to treat *P. aeruginosa* infections, the objective of this study was to describe susceptibility profiles of first line beta-lactam antimicrobials against *P. aeruginosa* at our academic medical center.

**Methods:**

All non-duplicate *P. aeruginosa* isolates from 2021-2023 were analyzed. First line beta-lactams evaluated included aztreonam (AZT), ceftazidime (CAZ), cefepime (CFP), piperacillin/tazobactam (PTZ), meropenem (MEM) as well as newer beta-lactams ceftolozane-tazobactam (C/T), and ceftazidime/avibactam (C/A). Susceptibility testing was performed with the BD Phoenix™ automated system or E-test. Data obtained for each patient included culture date, source of infection, hospital location, and MIC. Each MIC was categorized as susceptible, intermediate, or resistant based on 2024 CLSI MIC breakpoints.

**Results:**

Overall susceptibility rates for each beta-lactam for 1177 isolates were as follows: AZT (75%), CAZ (82%), CFP (83%), PTZ (79%), MEM (84%), C/T (99%), and C/A (99%). Moreover, 70% of *P. aeruginosa* strains were susceptible to all first line beta-lactams tested, while 25% displayed non-susceptibility to at least 1 first line beta-lactam, and 5% exhibited non-susceptibility to all first line beta-lactams tested. Among strains non-susceptible to at least 1 first line beta-lactam, the majority (37%) exhibited non-susceptibility to only 1 beta-lactam, while 20% and 19% displayed non-susceptibility to 2 or 3 beta-lactams, respectively. Additionally, 25% demonstrated non-susceptibility to 4 beta-lactams. Finally, respiratory isolates consistently displayed the highest incidence of non-susceptibility for each beta-lactam compared to blood, urine, wound, and miscellaneous body sites.

**Conclusion:**

This study highlights the variable susceptibility of first-line beta-lactam antimicrobials against *P. aeruginosa* infections. These findings stress the importance of customized treatment strategies, surveillance for evolving resistance, and considering newer beta-lactams such as ceftolozane-tazobactam and ceftazidime-avibactam, alongside prudent antibiotic utilization in clinical practice.

**Disclosures:**

**All Authors**: No reported disclosures

